# Unraveling the SES-science performance link: the distinct roles of peer closeness and conflict in primary and middle school

**DOI:** 10.3389/fpsyg.2026.1751063

**Published:** 2026-01-29

**Authors:** Zhi Liu, Jian Liu, Tianxue Cui

**Affiliations:** 1 Collaborative Innovation Center of Assessment for Basic Education Quality, Beijing Normal University, Beijing, China; 2 China Education Innovation Institute, Beijing Normal University, Zhuhai, China; 3 College of Public Administration and Humanities, Dalian Maritime University, Dalian, China

**Keywords:** peer closeness, peer conflict, primary and middle school students, science performance, socioeconomic status

## Abstract

**Introduction:**

This study proposed a moderation model to examine the moderating effect of peer closeness and conflict on the relationship between socioeconomic status (SES) and science performance among Chinese primary and middle school students. Peer closeness and peer conflict are conceptualized as social–relational contexts that may condition how family socioeconomic resources are translated into students’ science learning outcomes.

**Methods:**

A total of 19,108 4th graders and 9,983 8th graders completed online questionnaires and a standardized science test.

**Results:**

The moderation analysis indicated that peer closeness neither predicted students’ science performance nor moderated the association between SES and science performance for both grades. In contrast, peer conflict negatively predicted the science performance of 4th graders and moderated the association between SES and the science performance of 8th graders. Specifically, with a higher level of peer conflict, the positive relationship between SES and science performance was weaker.

**Discussion:**

The findings indicate that peer conflict is differentially associated with students’ science performance across grade levels. These findings highlight the distinct roles of positive and negative peer relationships in shaping science learning outcomes and underscore the importance of addressing peer conflict when promoting educational equity across different grade levels.

## Introduction

The rapid development of science and technology in the 21st century has increasingly underscored the importance of students’ scientific literacy and academic achievement in science. However, socioeconomic status (SES) disparities remain a significant obstacle to achieving equity in science education (OECD, 2016). Numerous studies have demonstrated a significant positive correlation between SES and students’ science achievement (Liu et al., 2020). High-SES families typically provide abundant social capital (e.g., material resources, practical opportunities, and educational support), whereas students from low-SES families face greater academic challenges due to the lack of such resources (Sirin, 2005).

Nonetheless, students from less advantaged backgrounds may partially offset socioeconomic constraints when they have greater opportunities to participate in science learning in school contexts (Bae and Lai, 2020). This is particularly relevant in science learning, a subject heavily relying on group collaboration and hands-on activities, making it uniquely valuable for research (Turan, 2023). In science learning, positive peer relationships (e.g., peer closeness) can significantly enhance students’ academic performance through emotional support, knowledge sharing, and collaborative learning (Țepordei et al., 2023). Conversely, peer conflict can disrupt group cooperation, which is associated with emotional distress and reduced learning efficiency, as evidenced by meta-analytic findings (Wentzel et al., 2018). This is especially true in the context of collectivist cultures like China, where group harmony is highly emphasized (Huang and Asghar, 2018).

However, existing research has limitations. Few studies have differentiated peer relationships into positive and negative aspects to simultaneously examine the moderating roles of peer closeness and peer conflict in the relationship between SES and students’ academic performance. Peer closeness reflects positive interactions, such as emotional support, trust, and collaboration, which are crucial for academic success and psychological well-being (Gorrese and Ruggieri, 2013). On the other hand, peer conflict reflects negative interactions, such as disputes, competition, and confrontation, which may adversely affect students’ emotional regulation and academic performance (Denham and Brown, 2010; Sebanc et al., 2016). This categorization helps capture the independent characteristics of both positive and negative peer relationships, offering a more comprehensive perspective for research.

Moreover, there may be developmental differences in the mechanisms of peer relationships across grade levels (e.g., fourth grade versus eighth grade). Younger students tend to rely more on direct support from family and teachers, whereas adolescents increasingly depend on peer relationships for academic and emotional support (Denham and Brown, 2010; Sebanc et al., 2016). Therefore, this study aims to address the following questions: (a) Do peer closeness or peer conflict moderate the relationship between SES and science achievement among fourth- and eighth-grade students? (b) How do the mechanisms by which peer closeness and peer conflict influence science achievement differ between these two grade levels? By exploring these questions, this study seeks to provide practical guidance for educators and policymakers, assisting in the design of targeted interventions, such as promoting peer closeness or reducing peer conflict. These efforts aim to support students from different SES backgrounds, enhance their science learning, and ultimately improve students’ science achievement and the overall quality of science education.

## Literature review

### Theoretical foundation

Peer relationships and their influence on students’ academic learning can be grounded in complementary developmental and sociological perspectives, which together clarify why peer closeness and conflict may operate as meaningful contexts for understanding SES-related disparities in science performance. From a developmental viewpoint, Bronfenbrenner’s ecological systems theory positions peer interactions within students’ microsystems, emphasizing that daily peer experiences constitute an immediate environment through which learning opportunities and adjustment processes are shaped (Bronfenbrenner, 1979). In a similar vein, Vygotsky’s social development theory conceptualizes learning as inherently situated in social interaction, highlighting that knowledge construction is facilitated through dialogue, shared activity, and collaboration with others—processes that are particularly salient in science classrooms where group discussion and inquiry-based tasks are common (Vygotsky, 1978; Rogoff, 1990).

Together, these perspectives establish peer relationships as proximal contexts in which students encounter, interpret, and respond to instructional demands, thereby making peer closeness and conflict theoretically relevant to science learning.

The compensatory capital theory provides a theoretical foundation for understanding the moderating role of peer closeness/conflict in the relationship between socioeconomic status (SES) and science achievement. This theory is primarily used to explain how individuals can compensate for disadvantages through non-economic resources in unequal socioeconomic conditions. Coleman (1988) proposed that social capital (e.g., close peer relationships, family and community support) and cultural capital (e.g., educational opportunities, cultural background) are crucial for individual development. He further emphasized that different forms of capital can complement each other, particularly for individuals from lower socioeconomic backgrounds. In contexts of low SES, individuals can achieve higher academic success if they are able to access these compensatory resources. This provides strong theoretical support for exploring the role of social capital in moderating the impact of SES on academic achievement. For instance, despite poor family economic conditions, students who receive emotional support from peers (e.g., peer closeness) or cultural capital (e.g., quality educational resources) in school or the community can compensate for economic shortcomings and enhance academic performance. Conversely, students from better family environments may have more capital to foster academic success, in which case peer conflict may weaken this effect. Thus, based on the compensatory capital theory, this study investigates the moderating role of peer closeness/conflict in the relationship between SES and science achievement.

### The relationship between SES and science performance

Numerous studies have shown a significant positive correlation between socioeconomic status (SES) and students’ academic achievement (Liu et al., 2020; Sirin, 2005). High-SES families can provide abundant material resources, such as experimental equipment, extracurricular tutoring, and books. Additionally, high-SES contexts may also be associated with greater opportunities to engage in science learning, which can indirectly support students’ academic development (Bae and Lai, 2020). These resources not only help students gain academic advantages but also foster their interest and long-term engagement in learning. In contrast, low-SES families, due to economic constraints, often lack the ability to provide abundant learning resources and typically have a family environment that does not sufficiently support science learning (Betancur et al., 2018).

Science subjects, with their unique characteristics, further amplify the impact of SES on academic achievement. Science learning often requires access to experimental resources and practical experience, which depend heavily on material support from both family and school. High-SES families can offer more opportunities for hands-on practice and better laboratory facilities, enabling students to gain a deeper understanding of scientific concepts. Additionally, science subjects emphasize logical reasoning and critical thinking, which require students to develop strong study habits and autonomous learning skills. However, students from low-SES families may lack such support, leading to greater challenges in their studies. Finally, parents from high-SES families are more likely to expose their children to a broader range of scientific content, which helps broaden their scientific perspective and contributes to improved science achievement. Therefore, SES not only influences access to learning resources but also shapes students’ thinking and scientific perspectives through family cultural capital.

### The relationship between peer relationships and science performance

Scientific learning often depends on collaboration and teamwork, such as group experiments, discussions, and project-based learning. As a result, peer relationships play a crucial role in academic performance, directly or indirectly influencing students’ learning outcomes by promoting cooperative learning and team collaboration (Wentzel, 1999). In science subjects, group discussions and experimental tasks require active interaction and cooperation among peers, and positive peer relationships can significantly enhance learning efficiency and academic achievement (Roseth et al., 2008; Johnson and Johnson, 2009).

Research has shown that peer closeness not only provides emotional encouragement but also helps students better understand complex scientific concepts through knowledge sharing (Endedijk and Breeman, 2022; Lester and Cross, 2015; Molinillo et al., 2018). In collaborative learning environments, peer closeness can enhance students’ emotional security and academic confidence, fostering greater engagement and performance in science (Wentzel and Watkins, 2002). However, peer conflict can have a significant negative impact on science achievement. Peer conflict often leads to emotional distress and distracted attention, which weakens students’ motivation and participation. In group tasks, conflict can damage trust and communication, reducing the effectiveness of teamwork and negatively impacting academic performance (Ladd and Kochenderfer-Ladd, 2009).

In the Chinese cultural context, the importance of peer relationships in academic performance is especially pronounced. A collectivist culture emphasizes interpersonal harmony and cooperative learning, making peer support and closeness an important social resource (Nguyen et al., 2009). In this environment, students prioritize collective harmony and mutual support, and positive peer relationships can effectively promote science learning and help compensate for low-SES disadvantages (Bae and Lai, 2020; Lan and Wang, 2024; Nguyen et al., 2009). However, this culture also intensifies the negative effects of peer conflict, as conflict is seen as a threat to group harmony, which can have a more pronounced detrimental impact on students’ emotions and academic outcomes (Nguyen et al., 2009). When peer conflict threatens group harmony, students may experience emotional distress, affecting both classroom engagement and academic performance.

### The moderating role of peer relationships in SES and science learning

Peer relationships, especially peer closeness and peer conflict, may play a crucial moderating role in the relationship between socioeconomic status (SES) and science achievement. High-SES families can provide students with abundant resources that support academic achievement. However, the quality of peer relationships can also influence the strength of the SES-academic performance link. Peer closeness can enhance students’ emotional security and academic confidence, which boosts their engagement and performance in science (Ladd and Kochenderfer-Ladd, 2009). For students from low-SES backgrounds, peer closeness is especially important because it can help compensate for the lack of academic and emotional support from their families (Coleman, 1988). In the context of science learning, peer closeness fosters greater participation in group discussions and projects, facilitating a deeper understanding of complex scientific concepts (Ladd and Kochenderfer-Ladd, 2009). Thus, the support from positive peer relationships can offset some of the academic challenges faced by students from low-SES families, potentially enhancing their science achievement.

On the other hand, while students from low-SES backgrounds may already face significant academic challenges, peer conflict can have an even more detrimental impact on high-SES students. This is because high-SES students tend to have higher academic expectations and place greater reliance on teamwork. These students, who often have better access to academic resources and are accustomed to collaborative and structured environments, may experience a more pronounced decline in performance when conflict disrupts their group dynamics. The breakdown of trust and communication in such situations can disproportionately hinder their academic success (Wentzel et al., 2018), as they may have developed more sophisticated interpersonal expectations and rely more heavily on group cohesion to achieve their goals.

In the Chinese context, collectivism emphasizes group harmony, making peer conflict even more detrimental to students’ emotional well-being and academic performance. When peer conflict threatens group harmony, students may experience significant emotional distress, which further impairs their engagement and academic achievement (Wentzel et al., 2018). Therefore, a moderator effect of peer relationships may exist on the relationship between SES and science achievement, where peer closeness can serve as a buffer, protecting low-SES students from the negative effects of limited family resources, whereas peer conflict can undermine this protective effect and exacerbate the academic challenges these students from high-SES families face.

### The present research

The above evidence has highlighted the crucial role of peer relationships in students’ SES and academic performance. However, existing research still has deficiencies that are worth further exploration. Therefore, this study focuses on the following three aspects: (a) Distinguishing between peer closeness and peer conflict to explore whether there are differences in their moderating mechanisms between SES and academic performance. (b) Focusing on specific academic subjects, science, to explore the importance of interpersonal relationships in science learning. Science education often involves collaborative projects, group experiments, and discussions that require effective communication and teamwork. These interactions help students to develop critical thinking skills, learn from diverse perspectives, and enhance their understanding of scientific concepts. Furthermore, positive peer interactions can foster a supportive learning environment, encourage curiosity, and increase student engagement in science activities, which are essential for academic success in this subject. (c) Investigating whether the mechanisms of peer relationships vary across different grade level groups of students. This study selects fourth-grade and eighth-grade students to explore the relationship between SES, peer relationships, and science achievement. There are notable differences between fourth-grade and eighth-grade students in terms of cognitive development, needs, and challenges. Fourth-grade students’ science learning is more focused on exploration and intuition, and at this stage, students rely more on family resources and teacher guidance (Furtak and Penuel, 2019). Peer relationships at this stage primarily play a role in collaboration to accomplish tasks. In contrast, eighth-grade students’ science learning emphasizes theoretical and logical reasoning skills. Additionally, with the onset of adolescence, students’ social interaction needs significantly increase, leading to a greater dependence on peer relationships (Liu et al., 2020). Adolescents are more sensitive to peer conflict, which can have a more disruptive impact on the learning environment and academic performance (Steinberg and Morris, 2001). In this stage, peer closeness is particularly important as it provides academic support and emotional stability to help students cope with more complex science learning tasks. Through a comparison of fourth and eighth-grade students, this study aims to explore the role of peer relationships in different grade levels and their impact on academic achievement.

### Hypothesis development

Extensive research has consistently demonstrated a positive association between socioeconomic status (SES) and students’ academic achievement, including science performance (Sirin, 2005; Liu et al., 2020). Science learning often requires access to inquiry-based experiences and instructional resources, which makes science achievement particularly sensitive to socioeconomic disparities (Betancur et al., 2018). Accordingly, the present study expects SES to be positively associated with students’ science performance.

*Hypothesis 1 (H1)*. Students’ socioeconomic status is positively associated with their science performance.

Drawing on social capital theory, social relationships within school contexts may condition how family-based resources are translated into academic outcomes (Coleman, 1988). Supportive peer relationships, reflected in peer closeness, have been linked to greater academic engagement and collaborative learning, which may partially compensate for socioeconomic disadvantages (Gorrese and Ruggieri, 2013; Endedijk and Breeman, 2022). Therefore, peer closeness is expected to moderate the association between SES and science performance.

*Hypothesis 2 (H2)*. Peer closeness moderates the association between SES and science performance.

In contrast, negative peer interactions may undermine students’ engagement and disrupt collaborative learning processes, thereby constraining the effective use of learning resources (Ladd and Kochenderfer-Ladd, 2009; Wentzel et al., 2018). Consequently, higher levels of peer conflict may weaken the positive association between SES and science performance.

*Hypothesis 3 (H3)*. Peer conflict moderates the association between SES and science performance.

Finally, developmental research suggests that the salience of peer relationships increases from childhood to adolescence, implying potential grade-level differences in peer effects (Sebanc et al., 2016; Steinberg and Morris, 2001). Therefore, the present study examines whether the hypothesized moderation effects differ between fourth- and eighth-grade students.

*Hypothesis 4 (H4)*. The moderating effects of peer closeness and peer conflict differ across grade levels.

## Methods

### Participants

We utilized two independent datasets for this study, both sourced from a large-scale anonymous assessment conducted by the author’s affiliated institution in China in 2022. A total of 19,353 fourth graders and 10,181 eighth graders participated in the online questionnaires and the hard copy of the subject test in science, with missing data rates of 1.27 and 1.94%, respectively. After excluding cases with missing data, the final analytic sample consisted of 19,108 fourth graders and 9,983 eighth graders. The proportion of female students was 49.80% in the fourth and 50.30% in the eighth grades.

### Instruments

Table 1 presents the reliability and psychometric properties of the scales for each student sample. Confirmatory factor analysis (CFA) was conducted for all multi-item scales, and the model fit indices (CFI, TLI, SRMR, and RMSEA) are reported in Table 1.

**Table 1:** Descriptive statistics, the psychometric properties and correlations among variables.

Variable	α	CFI	TLI	SRMR	RMSEA	Mean	SD	1	2	3	4
Grade 4 sample											
1. SES	–					−0.12	0.78	–			
2. Peer relationship	0.85	0.96	0.95	0.02	0.05	3.54	0.52	0.10^*^	–		
3. Peer closeness	0.83					3.53	0.59	0.09^*^	0.84^*^	–	
4. Peer conflict	0.81					1.46	0.63	−0.08^*^	−0.86^*^	−0.44^*^	--
5. Science achievement	--					500.00	100.00	0.28^*^	0.10^*^	0.07^*^	−0.10^*^
Grade 8 sample											
1. SES	–					−0.14	0.77	–			
2. Peer relationship	0.87	0.95	0.95	0.03	0.06	3.26	0.55	0.11^*^	–		
3. Peer closeness	0.89				.	3.23	0.66	0.13^*^	0.85^*^	–	
4. Peer conflict	0.82					1.71	0.64	−0.06^*^	−0.85^*^	−0.45^*^	–
5. Science achievement	–					500.00	100.00	0.27^*^	0.06^*^	0.06^*^	−0.05^*^

CFI, Comparative Fit index; TLI, Tucker-Lewis index; SRMR, Standardized root mean square residual; RMSEA, Root mean squared error of approximation. *p < 0.001.

#### Socioeconomic status (SES)

We constructed a composite SES index to assess students’ family socio-economic status (SES), following the methodology used in the Programme for International Student Assessment (PISA) (OECD, 2012). This index was created by averaging the standardized scores of three family-related variables: the highest parental occupation, the highest level of parental education, and family possessions. The parents’ occupational status was classified according to the “occupational prestige measures and socioeconomic index” in Li’s (2005, p. 90) study. Parental education levels were divided into six categories: *primary school, middle school, high school, junior college, college, and postgraduate (Master’s or Ph. D.)*. Family possessions were measured with six yes/no questions (*1 = yes; 0 = no*). The data for the latter two variables were derived from PISA 2009 (OECD, 2012).

This composite SES measure has been widely used in large-scale educational assessments and has demonstrated good reliability and validity in Chinese primary and middle school student samples (Ma et al., 2025; Cui et al., 2023). In addition, the indicators are concrete and familiar to students, facilitating accurate self-reporting and reducing potential measurement bias. Higher values on the SES index indicate more advantaged family backgrounds and greater access to educational resources.

#### Peer relationships

We employed the Peer Relationship Scale to assess the levels of closeness and conflict among peers within the school environment. This scale was developed by the National Children’s Study of China (NCSC) and has been frequently utilized in large-scale assessments in Mainland China (Dong and Lin, 2011). Students responded to ten items on a 4-point Likert scale, ranging from 1 (*Strongly Disagree*) to 4 (*Strongly Agree*), to indicate their peer interactions at school. Each domain, closeness and conflict, was measured with five items. Examples of these items included: “My classmates like me very much” (closeness) and “I often have disputes with my classmates” (conflict). A higher average score in each domain signified a greater perception of closeness or conflict with peers among the students.

The reliability and validity of the instruments are presented in Table 1. The peer relationship scales used in this study have been widely applied in prior research with Chinese student samples and have demonstrated acceptable reliability and construct validity (Cui et al., 2025). In the present study, internal consistency was satisfactory for both peer closeness and peer conflict across grade levels, indicating that the scales were suitable for subsequent analyses.

#### Academic achievement in science

The current study builds on the approach of Wu et al. (2022) to evaluate students’ science performance using a unified standardized test aligned with the National Curriculum Standards. This competency-based test adopts a three-dimensional framework encompassing knowledge (physics, biology, and geography), cognition (knowing, understanding, and applying), and scientific inquiry skills (questioning, seeking evidence, and explaining). Most items are contextualized within real-world scientific issues, reflecting students’ daily lives. The elementary science test consists of 27 items, including 25 multiple-choice questions and 2 open-ended questions, with a Cronbach’s alpha of 0.85. The middle school science test comprises 30 items, including 24 multiple-choice questions and 6 open-ended questions, with a Cronbach’s alpha of 0.88. A partial credit scoring approach is applied to the open-ended items. The resulting IRT scores are transformed into a standardized scale with a mean of 500 and a standard deviation of 100.

All instruments were administered in Chinese and have been previously used in comparable cultural and educational contexts, which helps to reduce potential linguistic or cultural bias in students’ interpretation of the survey items.

### Data collection procedures

The large-scale anonymous assessment was initially approved by the author’s affiliated institution and the local education authorities in the targeted area. The student evaluations began in October 2022 and lasted approximately 1 week. Stratified sampling was employed to recruit fourth and eighth graders, taking two variables into account: school type (public or private), school location (city, town, rural). With the approval of participating primary and middle schools, parents, and the students themselves, the students completed an online questionnaire at their convenience within the week. They also completed a standardized science test in accordance with the curriculum standards for the science subjects of the school period at a uniform time. Subsequent analyses were conducted separately for both samples to assess the replicability of the results across different grade levels.

### Data analytic procedures

We implemented Harman’s single-factor test to evaluate common method bias. The first common factor explained less than 40% of the variance, suggesting that the samples were not significantly affected by common method biases (Podsakoff et al., 2003). Preliminary analyses were conducted using SPSS 22.0 and Mplus 8.4, which included assessing the psychometric properties of the scales and descriptive statistics for all variables in both samples. To test H1, Pearson correlations were calculated to examine the relationship between socioeconomic status (SES) and science performance. According to Cohen (1992), Pearson correlation coefficients around 0.10 indicate a small effect size, around 0.30 indicate a moderate effect size, and above 0.50 indicate a strong effect size.

**Figure 1:**
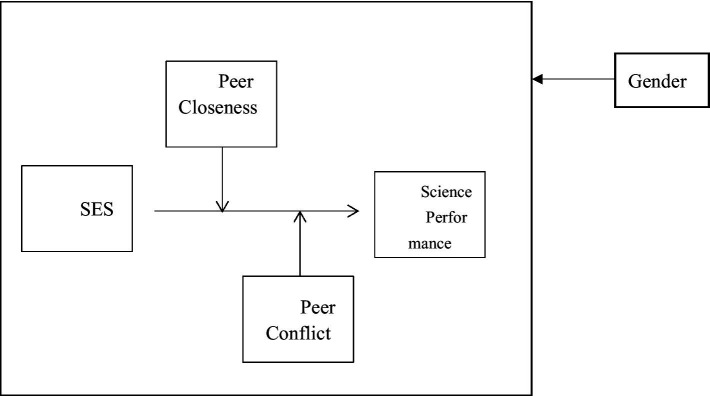
Conceptual model. The moderation effect of peer relationships (closeness and conflict) on the relationship between SES and science performance was examined while controlling for gender.

For H2 and H3, Model 2 of PROCESS 3.5.3 (Hayes, 2018) was utilized to test the moderating effects of peer closeness and conflict on the relationship between SES and science performance. Gender was included as a control variable in all regression analyses, given its well-established association with students’ academic achievement. Predictors were mean-centered to mitigate multicollinearity in the moderation model. PROCESS offers the advantage of automatically generating bootstrap confidence intervals, which helps to address potential non-normality in the sampling distribution. To test H4, the same analyses were replicated in both student samples. The overall conceptual model is presented in Figure 1.

## Results

### Preliminary analyses

#### Common method bias test

The Harman single-factor test results revealed that there were four factors with eigenvalues greater than 1 for both grade levels. Specifically, the 20 items accounted for 50.30% of the total variance in the model for 4th graders and 55.59% in the model for 8th graders. The first unrotated factor explained 23.20% of the variance for Grade 4 and 23.06% for Grade 8, both of which are below the 40% threshold suggested by Harman (1967). Consequently, these findings indicated that common method biases were not significant, ensuring the validity of the subsequent statistical analyses.

#### Descriptive statistics and correlations

The descriptive statistics and variable correlations are displayed in Table 1. All variables showed statistically significant correlations in each sample (*p*s < 0.001). Specifically, SES exhibited significant correlations with science performance in both samples, with correlation coefficients ranging from 0.27 to 0.28, indicating moderate effect sizes. This supported H1. Additionally, SES was significantly correlated with peer relationships, with correlation coefficients ranging from 0.10 to 0.11, reflecting small effect sizes. Peer relationships and their subdimensions were also significantly intercorrelated, with correlation coefficients ranging from −0.86 to 0.85, indicating large effect sizes.

### Association between SES and science performance (H1)

To examine Hypothesis 1, regression analyses were conducted to test whether SES was positively associated with students’ science performance. After controlling for gender, SES significantly predicted science performance in both fourth graders (B = 35.61, SE = 0.89, *p* < 0.001) and eighth graders (B = 34.57, SE = 1.26, *p* < 0.001). These results indicate that higher SES was associated with higher science performance across both grade levels, supporting Hypothesis 1.

### Moderating effects of peer closeness and peer conflict (H2 and H3)

**Figure 2:**
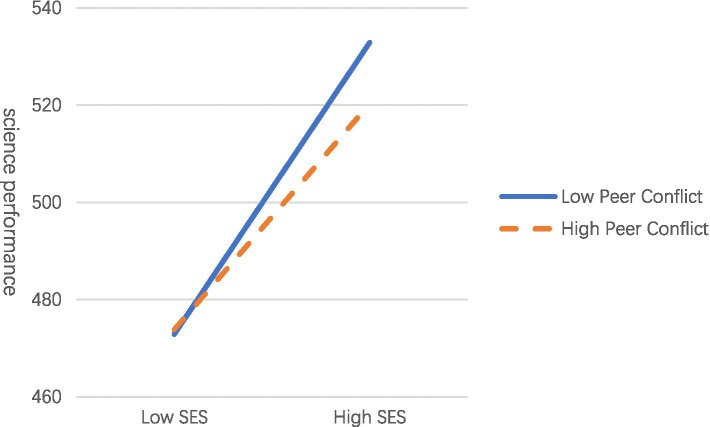
The moderating effect of the peer conflict for eighth graders. Low/high refers to below/above 1 SD of the target variable; the orange broken line represents a high level of peer conflict, and the blue solid line represents a low level of peer conflict.

Table 2 presents the findings for Hypotheses 2 and 3, examining the moderating effects of peer closeness and conflict on the relationship between SES and science performance. As shown in Table 2, the regression analyses report unstandardized coefficients (B), standard errors (SE), t values, 95% confidence intervals, and model R^2^ values for each grade-level model. After controlling for gender, SES positively predicted science performance in both fourth and eighth graders (Grade 4: *B* = 35.61, *SE* = 0.89; Grade 8: *B* = 34.57, *SE* = 1.26, *p*s < 0.001). Peer conflict negatively predicted science performance in both grades (Grade 4: *B* = −12.65, *SE* = 1.24; Grade 8: *B* = −4.53, *SE* = 1.68, *p*s < 0.01), whereas peer closeness did not significantly predict performance (*p*s > 0.19). Interaction terms between SES and peer closeness, as well as SES and peer conflict, did not significantly predict science performance in fourth graders, *B* = −1.33 and 0.04, *SE* = 1.65 and 1.56, *ps* > 0.42. However, a significant interaction between SES and peer conflict was observed in eighth graders (*B* = −6.83, *SE* = 2.01, *p* < 0.01). Simple effects analysis indicated that the relationship between SES and science performance was stronger for students with low peer conflict (*B* = 38.97, *p* < 0.001) compared to those with high peer conflict (*B* = 30.18, *p* < 0.001) (as detailed in Figure 2). These findings suggest that peer conflict moderates the relationship between SES and science performance for eighth graders but not for fourth graders, while peer closeness does not moderate this relationship for either grade. Consequently, H2 was not supported, H3 was partially supported (only for eighth graders).

**Table 2:** Moderating models for the association between socioeconomic status (SES), peer closeness, peer conflict, and science performance.

Predictors	Outcomes (science performance)							
	Grade 4 model (*n* = 19,108)				Grade 8 model (*n* = 9,983)			
	B	95%CIs	SE	t	B	95%CIs	SE	t
Constant	495.51	[493.58, 497.44]	0.98	504.39^***^	494.42	[491.74, 497.09]	1.36	362.29^***^
Gender	9.06	[6.34, 11.78]	1.39	6.53^***^	11.12	[7.33, 14.91]	1.93	5.75^***^
SES	35.61	[33.87, 37.36]	0.89	39.96^***^	34.57	[32.09, 37.05]	1.26	27.35^***^
PCL	1.70	[−0.87, 4.26]	1.31	1.30	1.22	[−2.03, 4.47]	1.66	0.74
PCF	−12.65	[−15.08, −10.22]	1.24	−10.21^***^	−4.53	[−7.82, −1.24]	1.68	−2.70^**^
PCL*SES	−1.33	[−4.55, 1.90]	1.65	−0.81	−2.43	[−6.26, 1.39]	1.85	−1.25
PCF*SES	0.04	[−3.01, 3.09]	1.56	0.03	−6.83	[−10.77, −2.88]	2.01	−3.39^**^
	*R^2^* = 0.19				*R^2^* = 0.38			

PCL, Peer Closeness; PCF, Peer Conflict. *p < 0.05, **p < 0.01, ***p < 0.001.

### Grade-level differences in moderation effects (H4)

Hypothesis 4 proposed that the moderating effects of peer closeness and peer conflict would differ across grade levels. The results showed that a significant moderation effect of peer conflict emerged only among eighth graders, whereas no moderation effects were observed among fourth graders. These findings suggest that the moderation patterns differed across grade levels; however, because peer closeness did not show moderation effects in either group and peer conflict moderated the SES–science performance relationship only in one grade, Hypothesis 4 was not supported.

## Discussion

This study explored the association between socioeconomic status (SES) and science performance and analyzed the moderating roles of peer conflict and peer closeness. The results showed that SES significantly predicted science performance, whereas peer closeness was not significantly associated with science test scores and did not moderate the relationship between SES and science performance. In contrast, the moderating effect of peer conflict in the relationship between SES and science performance differed across grade levels. No moderating effect was observed among fourth-grade students, whereas a significant moderation effect emerged among eighth-grade students. By distinguishing the different mechanisms of peer closeness and conflict, this study provides valuable insights for subsequent educational interventions in science education.

### SES and science performance (H1)

Consistent with Hypothesis 1, the present study found that socioeconomic status (SES) was significantly associated with students’ science performance in both fourth and eighth grades. This finding aligns with extensive prior research demonstrating persistent socioeconomic disparities in science achievement (Liu et al., 2020; OECD, 2016). High-SES families typically provide students with rich educational resources, such as science-related books and extracurricular activities (Barron et al., 2009; Betancur et al., 2018; Duncan and Murnane, 2011), which help students access a broader range of scientific knowledge and diverse academic perspectives. This diversity of knowledge and experiences may support students’ engagement with scientific content and contribute to higher levels of science performance. Furthermore, high-SES families not only offer material resources but also transmit social capital, such as the importance parents place on education, their educational background, and involvement, which may support students’ science learning (Lareau and Weininger, 2003; Archer et al., 2012). Parents’ understanding of scientific knowledge and their support for academic activities encourage students to participate more actively in class, fostering critical thinking and problem-solving skills.

### Absence of moderating effects of peer closeness (H2)

Although the quality of peer relationships is generally considered to have a positive impact on students’ psychological support and academic achievement (Țepordei et al., 2023), this study found that peer closeness did not significantly moderate the relationship between socioeconomic status (SES) and science performance, nor did it significantly predict students’ science scores. This finding may be related to the nature of science tasks, which are often goal-oriented and involve instrumental interactions that may limit the role of close peer relationships (Van Ryzin and Roseth, 2020). Specifically, as science tasks become more cognitively demanding in middle school, emotional support from peers may play a less direct role in influencing academic performance.

This result suggests that the task context plays a critical role in understanding the impact of peer relationships. Particularly in the case of demanding academic tasks, emotional support may not be as effectively translated into academic achievement, as the goal-oriented and instrumental nature of the tasks takes precedence. Therefore, in educational practice, it is important to focus on how task characteristics interact with students’ social interactions and affect academic performance.

### Direct and moderating effects of peer conflict (H3)

With respect to peer conflict, the results showed that peer conflict negatively predicted science performance in both fourth and eighth grades, with a stronger direct effect observed among fourth-grade students. This finding suggests that conflictual peer interactions may be associated with less favorable learning experiences and disruptions in classroom engagement, even at earlier stages of schooling.

In elementary school, science tasks are typically completed independently, and the classroom environment tends to be teacher-centered (Smit et al., 2018). Additionally, during elementary school, peer relationships may be less salient for academic functioning compared to later developmental stages (Denham and Brown, 2010). This developmental context may help explain why the interaction between peer conflict and SES was not significant, while a direct negative association between peer conflict and science performance was observed.

In contrast, during middle school, the complexity of science tasks increases significantly, and students tend to rely more on teamwork to complete assignments (Wentzel et al., 2018). At this stage, peer conflict may have a more complex association with science learning, as it can disrupt teamwork efficiency and be associated with differences in science performance. As students transition into adolescence, peer relationships become increasingly important, with the social focus shifting from parent–child relationships in the family context to peer interactions at school (Criss et al., 2016). Academic performance and learning behaviors are also increasingly shaped by peer relationships (Sebanc et al., 2016). This study provides empirical support for these ideas, demonstrating that peer conflict significantly negatively predicts science performance for both fourth- and eighth-grade students, with notable differences in the strength and mechanisms of this effect across these grade levels.

These findings suggest that the mechanisms through which peer conflict affects science learning differ across SES groups and grade levels. Specifically, in eighth grade, as adolescents experience more peer conflict, the moderating effect of SES becomes increasingly pronounced. This highlights the importance of educational practices that focus on developing students’ social skills and conflict resolution abilities to mitigate the negative impact of peer conflict on academic performance. Such interventions should be prioritized in diverse educational settings to foster a more supportive learning environment for all students.

### Grade-level differences in moderation effects (H4)

These findings are consistent with developmental perspectives suggesting that peer relationships become increasingly salient during adolescence, as students’ social orientation gradually shifts toward peer interactions (Sebanc et al., 2016; Steinberg and Morris, 2001). As a result, peer-related factors may play a more prominent role in shaping academic outcomes during middle school than in elementary school. The absence of moderation effects among fourth graders suggests that, at earlier developmental stages, family and teacher support may remain the primary determinants of academic performance in science.

### Limitations and future directions

This study only explored the potential moderating role of peer relationships between SES and science learning performance and analyzed the similarities and differences in the mechanisms between fourth-grade and eighth-grade students. In addition, although gender was controlled for in the analyses, other potential confounding variables—such as prior academic achievement, teacher quality, and school-level characteristics—were not included. These factors may also influence students’ science performance and peer relationships. The exclusion of these variables was primarily due to data availability constraints and the cross-sectional design of the study.

Moreover, the impact of peer relationships may differ across academic subject areas, whereas the present study focused exclusively on science learning. The sample was also limited to fourth- and eighth-grade students, and other developmental stages were not examined.

Future research could improve and expand in the following areas: further investigate the moderating role of peer relationships across different subject areas to understand their generality and specificity in various academic contexts; expand the research subjects to include other grade levels, particularly lower elementary and high school students, to comprehensively understand the role of peer relationships across different educational stages; adopt longitudinal research designs to track changes in the same cohort of students across different grades, providing insights into the long-term impact of peer relationships on the relationship between SES and academic performance; and conduct studies in different cultural contexts to explore the moderating role of peer relationships in various cultural settings, thereby enhancing the generalizability of the findings. Through these expansions, future research can more comprehensively reveal the complex mechanisms of peer relationships in the link between SES and student academic performance, providing more precise guidance for educational practice.

### Conclusion and practical implications

The current study examines the moderating roles of peer closeness and conflict in the association between SES and science performance among primary and middle school students. Specifically, after controlling for students’ gender, the study shows that peer closeness does not moderate the association between SES and science performance for either 4th or 8th graders. Additionally, peer closeness does not significantly predict science performance in Grades 4 and 8. In contrast, peer conflict moderates the association between SES and science performance among 8th graders, whereas it directly and negatively predicts science performance among 4th graders. These findings contribute to the literature by elucidating the distinct roles of peer closeness and peer conflict in shaping the association between SES and academic performance across various grade levels.

The findings of this study provide valuable insights into improving science performance among primary and middle school students, particularly in addressing the impact of peer relationships. First, schools should increase extracurricular science activities and experimental opportunities to address the resource deficits faced by students from low socioeconomic backgrounds, thus enhancing their science performance (Betancur et al., 2018). Secondly, since peer conflict moderates the relationship between socioeconomic status (SES) and science performance in 8th-grade students, schools should focus on strengthening conflict management. This can be achieved by implementing clearly defined group tasks to mitigate the negative impact of conflict on team efficiency (Van Ryzin and Roseth, 2020). Teachers should also take grade-level differences into account when designing science tasks. For younger students, designing more independent tasks can help reduce the severe interference of peer conflict on science achievement (Denham and Brown, 2010). In contrast, for 8th-grade students, optimizing group collaboration through clear role division and team rules is essential for enhancing teamwork effectiveness. Therefore, school administrators should adopt grade-specific strategies to address the unique needs of different age groups, addressing the negative associations between peer conflict and science performance, particularly among students with higher socioeconomic status.

Moreover, programs aimed at conflict resolution, such as conflict resolution workshops, peer mediation programs (Johnson and Johnson, 2009), and social–emotional learning (SEL) curriculums (Durlak et al., 2011), should be introduced to support students at all grade levels. By fostering foundational social skills in younger students and implementing more structured conflict management programs for older students, schools can better support students from diverse socioeconomic backgrounds, ultimately improving overall science education outcomes.

## Data Availability

The datasets presented in this article are not readily available. Requests to access the datasets should be directed to Tianxue Cui, YC07111@connect.um.edu.mo.
